# The Midwives Bill

**Published:** 1900-03-31

**Authors:** 


### The Hospital

A Journal of

The Medical Sciences and Hospital Administration.

Vol. XXVII.—No. 705.] Edited by Sir Henry Burdett, K.C.B., and Solomon C. Smith, M.D., M.R.C.P. [March 31, 1900.

### The Midwives Bill.

The discussion on the Midwives Bill now before Parliament, in the Standing Committee on Law of the House of Commons, which was reported in last Saturday's papers, appears to show that, even notwithstanding the amount of argument which has been expended upon the subject, there is anything but a clear idea, either among the opponents of the Bill or its supporters, of the real purposes which it ought to subserve, and of the conditions which it ought to fulfil. Colonel Milward said the Committee were probably hardly aware of the enormous change the Bill would make. There must now be some 23,000 persons acting in this way, and he was not prepared to go so far as to say that no person except a registered midwife should act in future. He thought the Committee ought not to go farther than to create a class of registered midwives. Mr. Heywood Johnstone, in reply, expressed considerable sympathy with the amendment, only objecting to it that it would leave untouched the case of the person who habitually and for gain practised midwifery, but who did not call herself a midwife; and, after some further unreported conversation, Colonel Milward expressed his readiness to withdraw his amendment, as he understood that his honourable friend was prepared to give benevolent consideration to the case of the persons whom it was intended to protect.

The only justification for the Bill, as it appears to us, and we must add that the justification is an ample one, is the necessity which exists to protect, not the 23,000 or any other number of ignorant women who may now attend cases of childbirth for gain, but the still larger number of thousands of quite poor mothers, who have hitherto been dependent upon the services of the 23,000 in question, with consequences which have been "writ large" in the reports of Coroners' inquests in all parts of the Kingdom, and to which the Coroners themselves have been assiduous in calling attention. In other words, the object of legislation should be to protect the lives of the poor, not to protect the industry of any persons, whether midwives or medical practitioners. In order to protect the lives of the poor, it is imperatively necessary to prohibit incompetent persons, whether they call themselves midwives or not, from attending cases of childbirth for gain. The frequent appearance of the trained nurse, in our streets and public conveyances, should not induce us to forget that representatives of the immortal "Sairey" are by no means difficult to discover in our slums. It is probable that the majority of Colonel Milward's 23,000 *protégés* have absolutely no knowledge at all of the process of parturition, of the dangers which it may entail, or of the symptoms which would point to the necessity of obtaining medical assistance, and the argument for legislation rests simply and entirely, as far as any national interests are concerned, upon the propriety of providing that no woman shall act as a responsible helper in a lying-in room unless she has given proofs of her fitness to stand between the parturient woman and the avoidable risks incidental to her condition. The General Medical Council has insisted again and again, in response to applications from successive ministries for its opinion upon this cardinal point, and has affirmed the necessity for legislation as a means of protecting the lives of mothers. The clause to which Colonel Milward took exception was introduced in order to meet the views of the Medical Council; and to soften down or abandon it would mean the abandonment of the Bill as a measure of public usefulness. Legislation has, no doubt, been advocated and promoted, in some quarters, less for the purpose of saving the lives of women in childbed than in order to constitute a new female professional class; and those who so regard the question would quite probably desire to enforce a high standard of attainment, and thus so far to "elevate" the members of the class in question that their services would hardly be available for the very poor, and those of Colonel Milward's 23,000 would still be required.

From the point of view of the public welfare this course of proceeding could not be defended, because it would fail to secure the results which at present are chiefly to be desired. In order to secure these results, it is above all things essential that the lowest existing class of practising midwives should be either taught or superseded; not merely prohibited from using the title, but prevented from doing the work. When this is accomplished, there will be nothing to hinder individual midwives from aiming at a higher standard than that which the law would require from them; but it is necessary to put a stop to the injuries inflicted by ignorance before we begin to speculate on the benefits which might be conferred by more advanced knowledge. Following the prophetic maxim, we must "cease to do evil" before we "learn to do well."

